# Supplementary material for: YTHDF2 governs muscle size through a targeted modulation of proteostasis
Source: Nat Commun. 2024 Mar 11;15:2176. doi: 10.1038/s41467-024-46546-8 (PMC10928198; doi:10.1038/s41467-024-46546-8)
Supplement: Supplementary file 1 — Supplementary Information [file 41467_2024_46546_MOESM1_ESM.pdf]

Supplementary Figure 1.

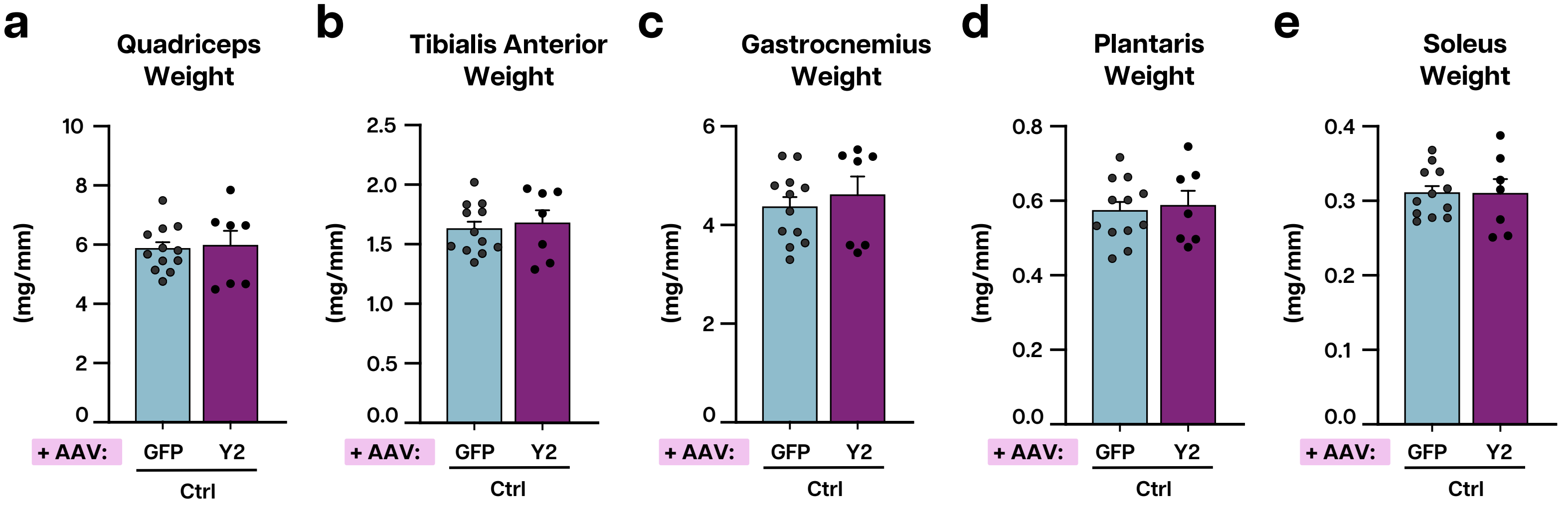

Supplementary Figure 1. Overexpression of YTHDF2 does not affect postnatal muscle size. (a) Quadriceps weight, (b) tibialis anterior weight, (c) gastrocnemius weight, (d) plantaris weight, and (e) soleus weight, normalized to tibia length for neonatal WT mice injected with AAV-GFP or AAV-YTHDF2 (AAV-Y2) as neonates and analyzed 2 months following administration. Biological animal replicates: n=12 (AAV-GFP) and 7 (AAV-Y2) for panel a-e. Data are presented as the mean ± SEM with the individual biological samples shown. Significance was determined by 2-sided Student's t-test for comparisons between AAV-GFP- and AAV-Y2-injected mice at 2 months.

Supplementary Figure 2.

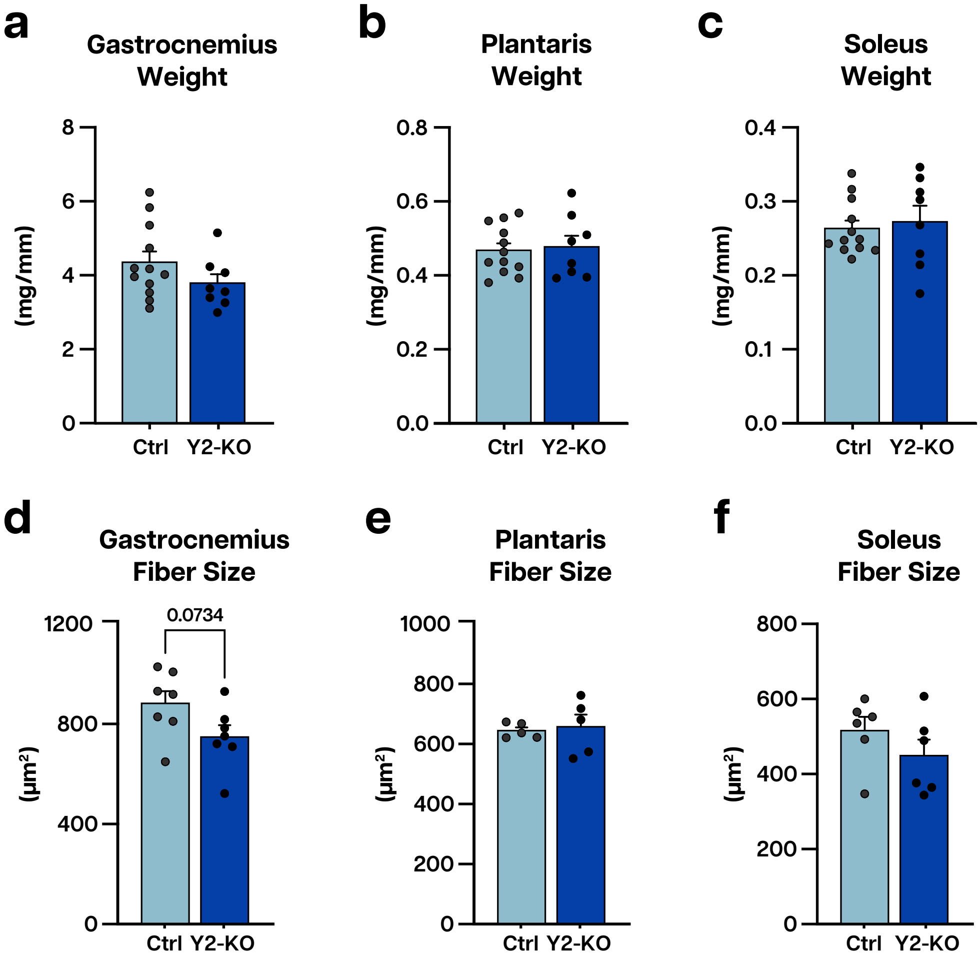

Supplementary Figure 2. Not all muscles are significantly impaired by two months of age in YTHDF2-null mice. (a) Gastrocnemius, (b) plantaris, and (c) soleus weight for Ctrl and Y2-KO mice at 2 months of age, normalized to tibia length. Myofiber cross-sectional areas for (d) gastrocnemius, (e) plantaris, and (f) soleus for Ctrl and Y2-KO mice at 1 and 2 months of age. Biological animal replicates: n=12 (Ctrl) and 8 (Y2-KO) for panel a-c; n=7 (Ctrl) and 7 (Y2-KO) for panel d; n=5 (Ctrl) and 5 (Y2-KO) for panel e; n=6 (Ctrl) and 6 (Y2-KO) for panel f. Data are presented as the mean ± SEM with the individual biological samples shown. Significance was determined by 2-sided Student's t-test for comparisons between Ctrl and Y2-KO mice at 2 months.

Supplementary Figure 3.

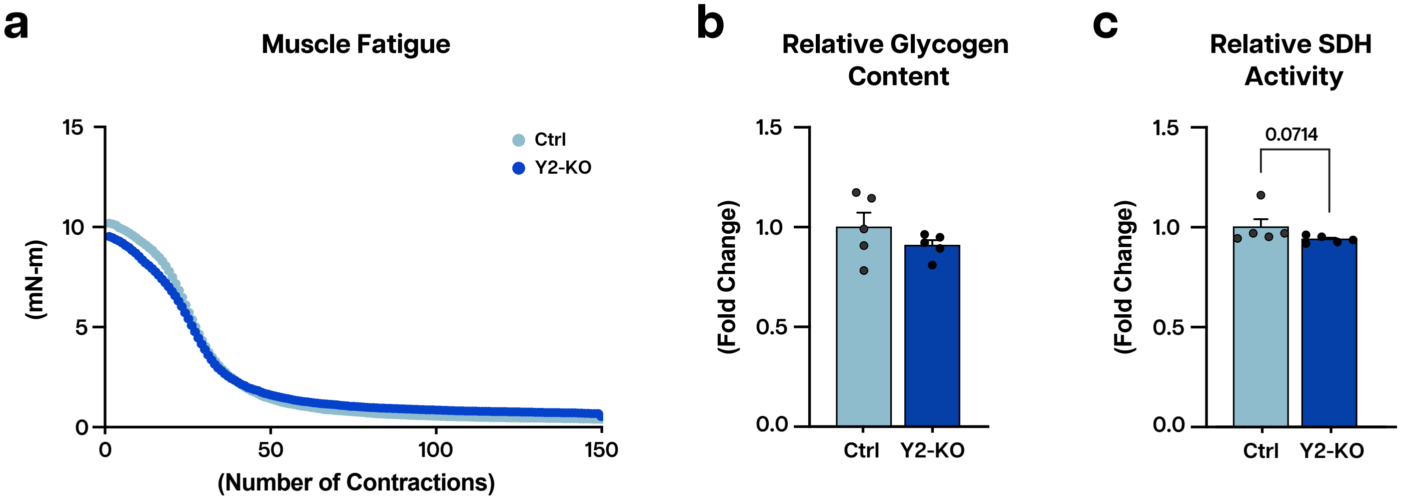

Supplementary Figure 3. Muscles lacking YTHDF2 show no significant changes in fatiguability, glycogen distribution, or oxidative capacity. (a) In vivo muscle twitch and tetanic torque measures for Ctrl and Y2-KO across a 5-minute fatigue resistance protocol (150 contractions). (b) Glycogen distribution and (c) succinate dehydrogenase activity, with input load normalized to muscle weight, as determined via colorimetric assay kits for Ctrl and Y2-KO quadriceps. Biological animal replicates: n=7 (Ctrl) and 7 (Y2-KO) in panel a; n=5 (Ctrl) and 5 (Y2-KO) in panel b and c. Data are presented as the mean ± SEM with the individual biological samples shown. Significance was determined by 2-sided Student's t-test for comparisons between Ctrl and Y2-KO mice.

Supplementary Figure 4.

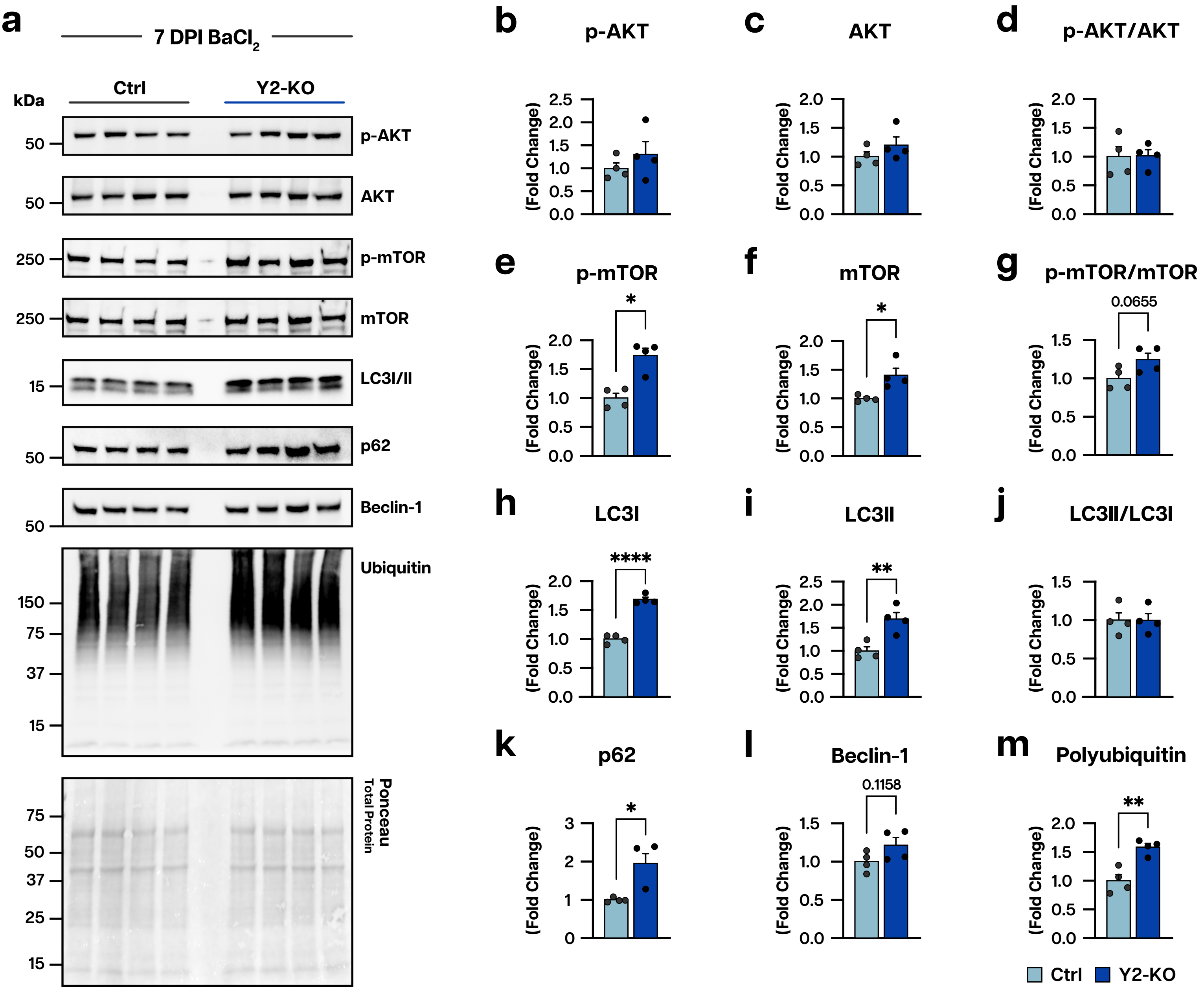

Supplementary Figure 4. The proteostasis signature of YTHDF2-null muscles is altered in acute injury. (a) Western blot analyses and quantification using total protein detection by Ponceau stain as a loading control for (b) phosphor-(p-)AKT, (c) AKT, (d) p-AKT/AKT ratio, (e) p-mTOR, (f) mTOR, (g) p-mTOR/AKT ratio, (h) LC3I, (i) LC3II, (j) LC3II/LC3I ratio, (k) p62, (l) Beclin-1, and (m) polyubiquitin in Ctrl and Y2-KO tibialis anterior 7 days post-injection with BaCl2. Biological animal replicates: n=4 (Ctrl) and 4 (Y2-KO) in panel a-m. Data are presented as the mean ± SEM with the individual biological samples shown. Significance was determined by 2-sided Student's t-test for comparisons between Ctrl and Y2-KO mice. Welch's correction was used for unequal variances: *p≤0.05, **p≤0.01, ****p≤0.0001.

Supplementary Figure 5.

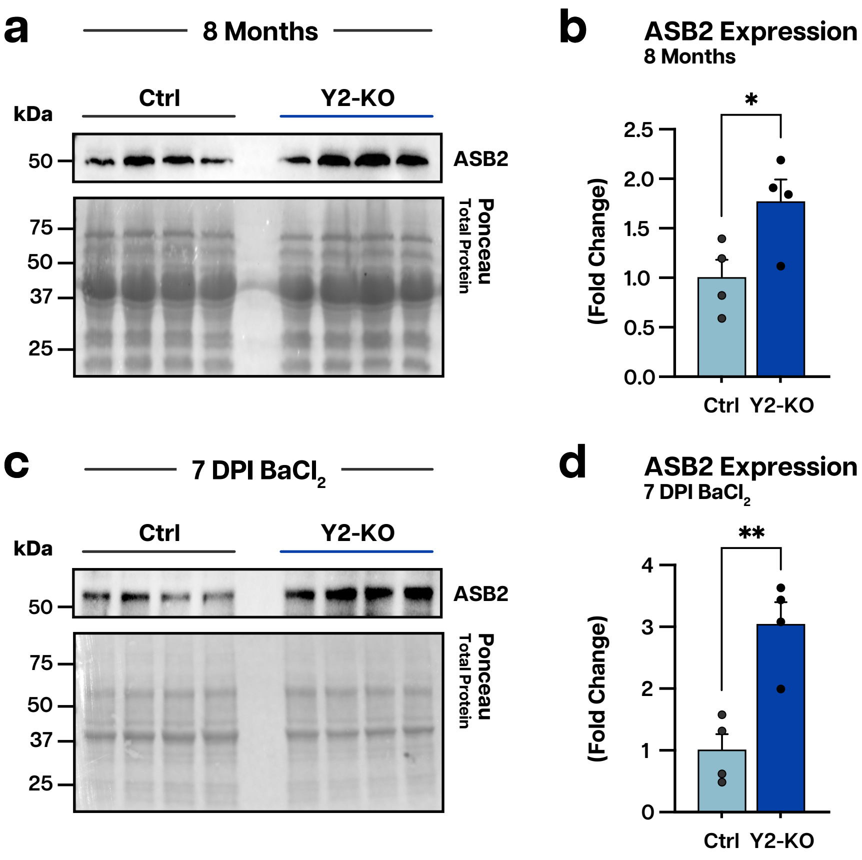

Supplementary Figure 5. YTHDF2-deficient muscles retain elevated ASB2 with aging and acute injury. (a) Western blot analyses and (b) quantification using total protein detection by Ponceau stain as a loading control for ASB2 expression in 8-month-old Ctrl and Y2-KO quadriceps. (c) Western blot analyses and (d) quantification using total protein detection by Ponceau stain as a loading control for ASB2 expression in Ctrl and Y2-KO tibialis anterior 7 days post-injection with BaCl2. Biological animal replicates: n=4 (Ctrl) and 4 (Y2-KO) in panel a-d. Data are presented as the mean ± SEM with the individual biological samples shown. Significance was determined by 2-sided Student's t-test for comparisons between Ctrl and Y2-KO mice: $*p \leq 0.05$, $**p \leq 0.01$.

Supplementary Figure 6.

a

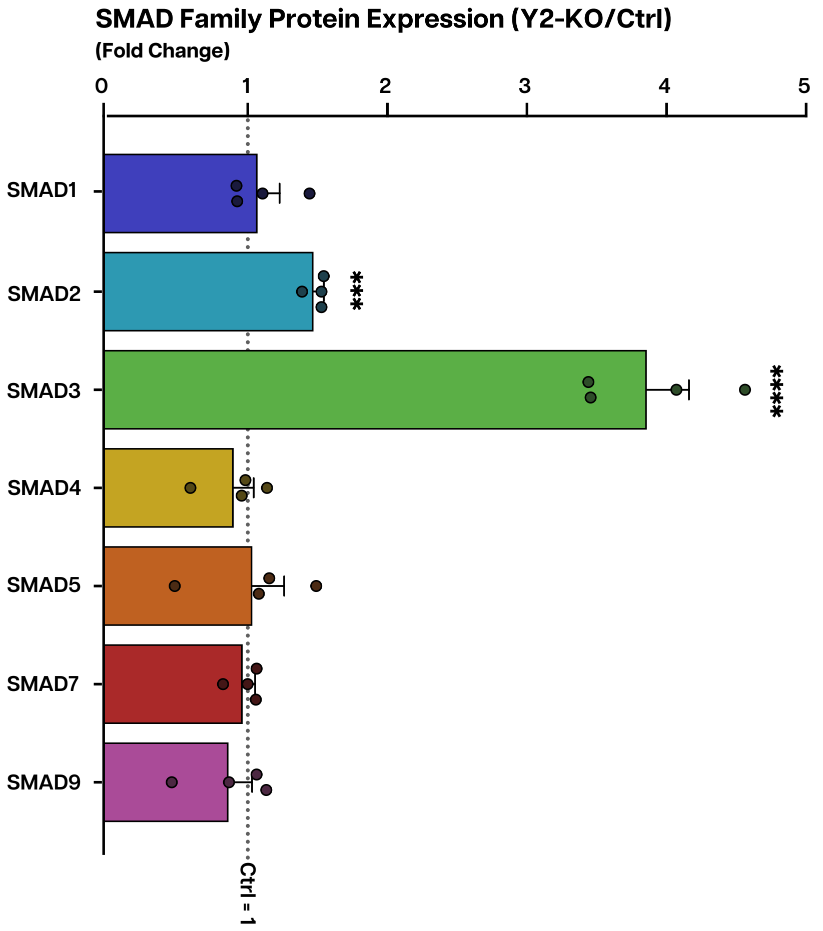

Supplementary Figure 6. YTHDF2 modulates antihypertrophic SMAD levels. (a) Quantification of SMAD proteins 1, 2, 3, 4, 5, 7, and 9 using total protein detection by (respective) Ponceau stain as a loading control in Ctrl and Y2-KO quadriceps at 2 months of age. Biological animal replicates: n=4 (Ctrl) and 4 (Y2-KO). Data are presented as the mean ± SEM with the individual biological samples shown. Significance was determined by 2-tailed Student's t-test for comparisons between Ctrl and Y2-KO mice: ***p≤0.001, ****p≤0.0001.

Supplementary Figure 7.

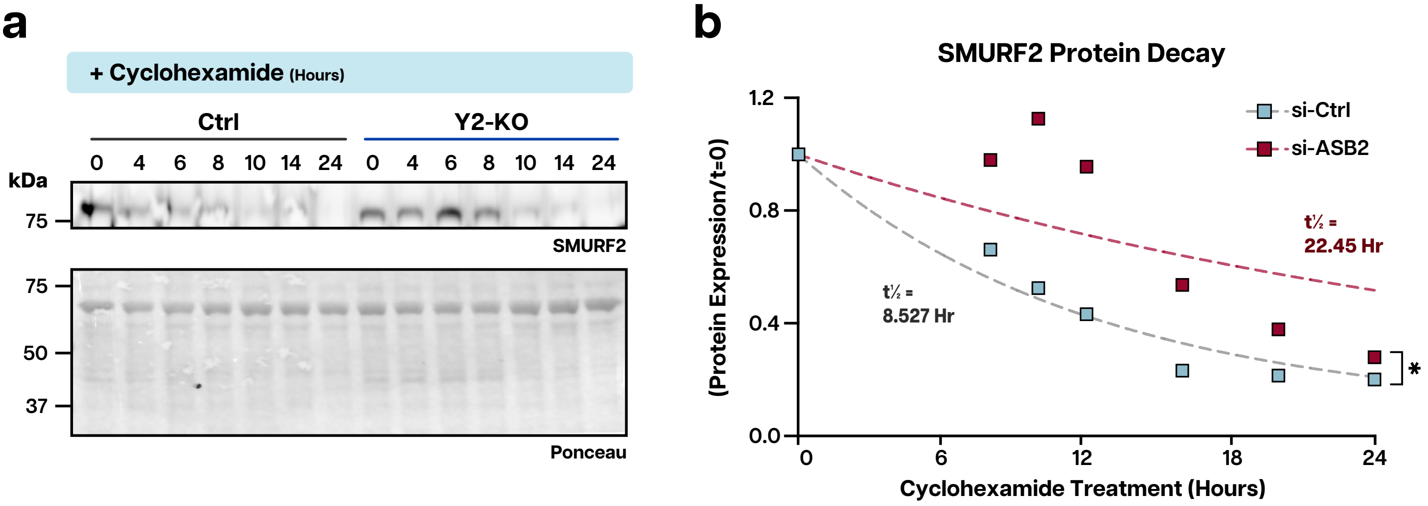

Supplementary Figure 7. ASB2 regulates SMURF2 protein stability. (a) Western blot analysis for SMURF2 and (b) quantification following cyclohexamide treatment for the indicated times in H9C2 rat myoblasts transfected with an siRNA pool targeting ASB2 or control non-targeting siRNA (si-Ctrl), normalized to respective t=0 values. Single cell replicates were used. Best-fit values for half-life and decay rate were calculated using least squares regression analysis: *$p \leq 0.05$.
