# Supplementary material for: YTHDF2 governs muscle size through a targeted modulation of proteostasis
Source: Nat Commun. 2024 Mar 11;15:2176. doi: 10.1038/s41467-024-46546-8 (PMC10928198; doi:10.1038/s41467-024-46546-8)
Supplement: Supplementary file 3 — Description of Additional Supplementary Files [file 41467_2024_46546_MOESM3_ESM.pdf]

Description of Additional Supplementary Files

Title: Supplementary Data 1

Description Diferentilly expressed proteins for Ctrl or Y2-KO skeletal muscles detected via TMT LC5 MS/MS.

Title: Supplementary Data 2

Description YTHDF2-bound transcripts detected by YTHDF2-RIP-seq analysis on skeletal muscle.

Title: Supplementary Data 3

Description Recorded signifcant p-values from statitcal tests.
