# Supplementary material for: YTHDF2 governs muscle size through a targeted modulation of proteostasis
Source: Nat Commun. 2024 Mar 11;15:2176. doi: 10.1038/s41467-024-46546-8 (PMC10928198; doi:10.1038/s41467-024-46546-8)
Supplement: Supplementary file 7 — Reporting Summary [file 41467_2024_46546_MOESM7_ESM.pdf]

nature portfolio

Corresponding author(s): Federica Accornero

Last updated by author(s): Jan 4, 2024

Reporting Summary

Nature Portfolio wishes to improve the reproducibility of the work that we publish. This form provides structure for consistency and transparency in reporting. For further information on Nature Portfolio policies, see our Editorial Policies and the Editorial Policy Checklist.

Statistics

For all statistical analyses, confirm that the following items are present in the figure legend, table legend, main text, or Methods section.

n/a	Confirmed	
☐	☒	The exact sample size (*n*) for each experimental group/condition, given as a discrete number and unit of measurement
☐	☒	A statement on whether measurements were taken from distinct samples or whether the same sample was measured repeatedly
☐	☒	The statistical test(s) used AND whether they are one- or two-sided<br>*Only common tests should be described solely by name; describe more complex techniques in the Methods section.*
☒	☐	A description of all covariates tested
☐	☒	A description of any assumptions or corrections, such as tests of normality and adjustment for multiple comparisons
☐	☒	A full description of the statistical parameters including central tendency (e.g. means) or other basic estimates (e.g. regression coefficient) AND variation (e.g. standard deviation) or associated estimates of uncertainty (e.g. confidence intervals)
☐	☒	For null hypothesis testing, the test statistic (e.g. *F*, *t*, *r*) with confidence intervals, effect sizes, degrees of freedom and *P* value noted<br>*Give P values as exact values whenever suitable.*
☒	☐	For Bayesian analysis, information on the choice of priors and Markov chain Monte Carlo settings
☒	☐	For hierarchical and complex designs, identification of the appropriate level for tests and full reporting of outcomes
☒	☐	Estimates of effect sizes (e.g. Cohen's *d*, Pearson's *r*), indicating how they were calculated

Our web collection on statistics for biologists contains articles on many of the points above.

Software and code

Policy information about availability of computer code

Data collection	Running tests were conducted via Comprehensive Lab Animal Monitoring System (CLAMS) with data collection using OxyMax software Ver 2.4.2 (Columbus Instruments, Columbus, OH, USA). In vivo muscle force was collected via 1300A 3-in-1 Whole Animal System for Mice, Model 1300A (Aurora Scientific). Image collection was performed using a EVOS Imaging System (Invitrogen, Thermo Fisher Scientific, Waltham, MA, USA) or using Zeiss AxioScope microscope and Zeiss AxioCam monochrome charge-coupled device (CCD) camera and AxioVision software SE64 Rel. 4.8 (ZEISS, Dublin, CA, USA). Western blots were imaged via ChemiDoc TOUCH Imaging System (BIO-RAD, Hercules, CA, USA). qPCR was performed via CFX Connect (BIO-RAD)
Data analysis	Image quantifications were conducted using ImageJ 1.53k (National Institutes of Health [NIH], Bethesda, MD, USA). ImageJ and Image Lab Ver 6.10 (BIO-RAD) were used for Western blot quantification. CFX Maestro (BIO-RAD) and Microsoft Excel Ver 16.72 (Microsoft, Redmond, WA, USA) were used for qPCR analysis. Graphing and statistical analyses were conducted using Graph Pad Prism Ver 9.4.0 (Graph Pad Software).

For manuscripts utilizing custom algorithms or software that are central to the research but not yet described in published literature, software must be made available to editors and reviewers. We strongly encourage code deposition in a community repository (e.g. GitHub). See the Nature Portfolio guidelines for submitting code & software for further information.

Data

Policy information about availability of data

All manuscripts must include a data availability statement. This statement should provide the following information, where applicable:

- Accession codes, unique identifiers, or web links for publicly available datasets
- A description of any restrictions on data availability
- For clinical datasets or third party data, please ensure that the statement adheres to our policy

Source data are provided with this paper. Proteomics data are available through MassIVE (https://doi.org/doi:10.25345/C56T0H66F). m6A sequencing analyses are available through Gene Expression Omnibus (https://www.ncbi.nlm.nih.gov/geo/query/acc.cgi?acc=GSE179368).

Research involving human participants, their data, or biological material

Policy information about studies with human participants or human data. See also policy information about sex, gender (identity/presentation), and sexual orientation and race, ethnicity and racism.

Reporting on sex and gender	n/a
Reporting on race, ethnicity, or other socially relevant groupings	n/a
Population characteristics	n/a
Recruitment	n/a
Ethics oversight	n/a

Note that full information on the approval of the study protocol must also be provided in the manuscript.

Field-specific reporting

Please select the one below that is the best fit for your research. If you are not sure, read the appropriate sections before making your selection.

☒ Life sciences ☐ Behavioural & social sciences ☐ Ecological, evolutionary & environmental sciences

For a reference copy of the document with all sections, see nature.com/documents/nr-reporting-summary-flat.pdf

Life sciences study design

All studies must disclose on these points even when the disclosure is negative.

Sample size	Sample sizes for animal studies were selected based on the power calculations referenced within the IACUC animal protocol deemed to be appropriate and sufficient for realization of statistical significance for in vivo experiments. For ex vivo quantifications, all cells per biological replicate were included in the analyses. For biochemical assessments, historical data from similar experiments performed by our lab informed sample size choices, given the rationale of having ~80% of power to detect a difference of 1.5-fold SD in the parameters of interest between 2 treatment groups (with a 2-sided a=5%).
Data exclusions	Grubbs' (ESD) tests were run using Graph Pad Prism to identify outliers. This resulted in exclusion of an outlier data point in two panels: forced running test and RNA decay assay, as indicated in the correspondent figure legends.
Replication	Experiments were successfully reproduced at least 3 times. Number of biological replicates in each panel is included in figure legends.
Randomization	Y2-KO mice were housed with their littermate controls. For all animal experiments, cages/mice were randomly allocated to study groups based on order they were tagged for identification. Age-matched Ctrl or Y2-KO mice were randomly assigned to sham and overload operations (and siRNA administration).
Blinding	Investigators were blinded to mouse genotypes when performing ablation surgeries, and cell measurements; gravimetric data was collected with individuals weighing samples while blinded to genotypes. Similarly, biochemical and bioinformatics approaches were performed in a blinded fashion. Unbiased approaches were implemented whenever possible and reasonable to limit investigator bias.

Reporting for specific materials, systems and methods

We require information from authors about some types of materials, experimental systems and methods used in many studies. Here, indicate whether each material, system or method listed is relevant to your study. If you are not sure if a list item applies to your research, read the appropriate section before selecting a response.

Materials & experimental systems

n/a	Involved in the study	
☐	☒	Antibodies
☐	☒	Eukaryotic cell lines
☒	☐	Palaeontology and archaeology
☐	☒	Animals and other organisms
☒	☐	Clinical data
☒	☐	Dual use research of concern
☒	☐	Plants

Methods

n/a	Involved in the study	
☒	☐	ChIP-seq
☒	☐	Flow cytometry
☒	☐	MRI-based neuroimaging

Antibodies

Antibodies used

YTHDF2 (abcam, #ab220163, 1:1000)
Myosin heavy chain Type I (DSHB, #BA-D5, 1:40)
Myosin heavy chain Type IIA (DSHB, #SC-71, 1:10)
Myosin heavy chain Type IIB (DSHB, #BF-F3, 1:40)
Myosin heavy chain [all but Type IIX] (DSHB, #BF-35, 1:40)
Goat anti-Mouse IgG2b Cross-Adsorbed Secondary Antibody, Alexa Fluor 647 (Invitrogen, #A-21242, 1:1000)
Goat anti-Mouse IgG1 Cross-Adsorbed Secondary Antibody, Alexa Fluor 647 (Invitrogen, #A-21240, 1:1000)
Goat anti-Mouse IgM (Heavy chain) Secondary Antibody, Alexa Fluor 647 (Invitrogen, #A-21238 1:1000)
Laminin (Millipore Sigma, #L9393, 1:1000)
Desmin (Thermo Fisher Scientific, #PA5-16705, 1:200)
Goat anti-Rabbit IgG Secondary Antibody, DyLight 488 (Thermo Fisher Scientific, #35552, 1:1000)
ASB2 (Invitrogen, #PA5-29476, 1:500)
SMAD1 (Cell Signaling Technology, D95D7, #6944, 1:1000)
SMAD2 (Cell Signaling Technology, D43B4, #5339, 1:1000)
SMAD3 (Cell Signaling Technology, C67H9, #9523, 1:1000)
SMAD4 (Cell Signaling Technology, D3M6U, #38454, 1:1000)
SMAD5 (Cell Signaling Technology, D4G2, #12534, 1:1000)
SMAD7 (abcam, #ab216428, 1:1000)
SMAD9 (abcam, #ab96698, 1:1000)
SMURF2 (Cell Signaling Technology, D8B8, #12024, 1:1000)
phosphor-SMAD3 (abcam, ab52903, 1:1000)
FOXO3a (Cell Signaling Technology, D19A7, #12829, 1:1000)
phosphor-mTOR (Cell Signaling Technology, D9C2, #5536, 1:1000)
mTOR (Cell Signaling Technology, 7C10, #2983, 1:1000)
phosphor-AKT (Cell Signaling Technology, D9E, #4060, 1:1000)
AKT (Cell Signaling Technology, #9272, 1:1000)
LC3I/II (Cell Signaling Technology, D3U4C, #12741 1:1000)
Beclin-1 Cell Signaling Technology, D40C5, #3495, 1:1000)
p62 (Cell Signaling Technology, D6M5X, #23214, 1:1000)
Ubiquitin (Cell Signaling Technology, E6K4Y, #20326, 1:1000)

Validation

All below primary antibodies have been validated for their use(s) per manufacturer site and publication references.
YTHDF2 (https://www.abcam.com/products/primary-antibodies/ythdf2-antibody-epr20318-ab220163.html)
Myosin heavy chain Type I (https://dshb.biology.uiowa.edu/BA-D5)
Myosin heavy chain Type IIA (https://dshb.biology.uiowa.edu/SC-71)
Myosin heavy chain Type IIB (https://dshb.biology.uiowa.edu/BF-F3)
Myosin heavy chain [all but Type IIX] (https://dshb.biology.uiowa.edu/BF-35)
Laminin (https://www.sigmaaldrich.com/US/en/product/sigma/l9393)
Desmin (https://www.thermofisher.com/antibody/product/Desmin-Antibody-Polyclonal/PA5-16705)
ASB2 (https://www.thermofisher.com/antibody/product/ASB2-Antibody-Polyclonal/PA5-29476)
SMAD1 (https://www.cellsignal.com/products/primary-antibodies/smad1-d59d7-xp-rabbit-mab/6944)
SMAD2 (https://www.cellsignal.com/products/primary-antibodies/smad2-d43b4-xp-rabbit-mab/5339)
SMAD3 (https://www.cellsignal.com/products/primary-antibodies/smad3-c67h9-rabbit-mab/9523)
SMAD4 (https://www.cellsignal.com/products/primary-antibodies/smad4-d3m6u-rabbit-mab/38454)
SMAD5 (https://www.cellsignal.com/products/primary-antibodies/smad5-d4g2-rabbit-mab/12534)
SMAD7 (https://www.abcam.com/products/primary-antibodies/madh7smad7-antibody-ab216428.html)
SMAD9 (https://www.abcam.com/products/primary-antibodies/smad9-antibody-ab96698.html)
SMURF2 (https://www.cellsignal.com/products/primary-antibodies/smurf2-d8b8-rabbit-mab/12024)
phosphor-SMAD3 (https://www.abcam.com/products/primary-antibodies/smad3-phospho-s423--s425-antibody-ep823y-ab52903.html)
FOXO3a (https://www.cellsignal.com/products/primary-antibodies/foxo3a-d19a7-rabbit-mab/12829)
phosphor-mTOR (https://www.cellsignal.com/products/primary-antibodies/phospho-mtor-ser2448-d9c2-xp-174-rabbit-mab/5536)
mTOR (https://www.cellsignal.com/products/primary-antibodies/mtor-7c10-rabbit-mab/2983)
phosphor-AKT (https://www.cellsignal.com/products/primary-antibodies/phospho-akt-ser473-d9e-xp-rabbit-mab/4060)
AKT (https://www.cellsignal.com/products/primary-antibodies/akt-antibody/9272)
LC3I/II (https://www.cellsignal.com/products/primary-antibodies/lc3a-b-d3u4c-xp-rabbit-mab/12741)
Beclin-1 (https://www.cellsignal.com/products/primary-antibodies/beclin-1-d40c5-rabbit-mab/3495)
p62 (https://www.cellsignal.com/products/primary-antibodies/sqstm1-p62-d6m5x-rabbit-mab/23214)

Ubiquitin was validated through this publication, whereby monomeric and poly-ubiquitin were detected across all treated samples.

Eukaryotic cell lines

Policy information about cell lines and Sex and Gender in Research

Cell line source(s)	H9C2 rat myoblasts (ATCC, # CRL-1446) were used in this study.
Authentication	Cells were purchased from ATCC and not further authenticated.
Mycoplasma contamination	Cells were tested and confirmed negative for mycoplasma contamination.
Commonly misidentified lines (See ICLAC register)	No commonly misidentified lines were used in this study.

Animals and other research organisms

Policy information about studies involving animals; ARRIVE guidelines recommended for reporting animal research, and Sex and Gender in Research

Laboratory animals	Male and female C57BL6/N mice up to 8 months of age were used in this study. Ythdf2 LoxP-targeted (flox; fl) mice (Ythdf2fl/fl) were generated by Cyagen (Santa Clara, CA, USA). Ythdf2fl/fl mice were crossed with mice expressing Cre recombinase gene under the control of the skeletal muscle-specific Myogenin (MyoG) promoter to obtain muscle-restricted deletion of Ythdf2 (Y2-KO). Ythdf2fl/fl littermates not expressing Cre recombinase were used as controls. Mouse age is specified in each figure panel; genotypes were cohoused from the same litter (Ctrl/Y2-KO) and age-matched for studies. Animals were housed at 72°F (22°C) at 50% humidity under a 12-hour light/ 12-hour dark cycle with ad libitum access to standard chow diet and water.
Wild animals	This study did not involve wild animals.
Reporting on sex	All findings include both animal sexes in comparable numbers
Field-collected samples	This study did not include samples collected from the field.
Ethics oversight	All presented experiments comply with the standards set forth by the Institutional Animal Care and Use Committee at The Ohio State University, and the Guide and Care and Use of Laboratory Animals published by the US National Institute of Health. All procedures are approved by The Ohio State University Institutional Animal Care and Use Committee and Institutional Biosafety Committee under protocol 2015A00000115-R2.

Note that full information on the approval of the study protocol must also be provided in the manuscript.

Plants

Seed stocks	n/a
Novel plant genotypes	n/a
Authentication	n/a
